# Clinical and genetic characteristics of Chinese patients with congenital fibrosis of the extraocular muscles

**DOI:** 10.1186/s13023-024-03206-w

**Published:** 2024-08-15

**Authors:** Jin Wu, Lijuan Huang, Yunyu Zhou, Yan Xie, Tong Mo, Ningdong Li

**Affiliations:** 1 Department of Ophthalmology, Beijing Children’s Hospital, Capital Medical University, Beijing, 100045 China; 2 Department of Ophthalmology, Shenzhen Children’s Hospital, Shenzhen, 518031 China; 3 Department of Ophthalmology, The Second Affiliated Hospital of Fujian Medical University, Quanzhou, 362000 China; 4 Department of Radiology, Shenzhen Children’s Hospital, Shenzhen, 518031 China; 5 Department of Ophthalmology, Shanghai General Hospital, Shanghai, 200940 China

**Keywords:** Congenital fibrosis of the extraocular muscles, *KIF21A*, *TUBB3*, Mutation

## Abstract

**Objective:**

This study aimed to describe the clinical and genetic characteristics of Chinese patients with congenital fibrosis of the extraocular muscles (CFEOM), and to evaluate the phenotype–genotype correlations in these patients.

**Methods:**

This was a retrospective study. Patients with CFEOM underwent detailed ophthalmic examinations and magnetic resonance imaging (MRI). Panel-based next-generation sequencing was performed to identify pathogenic variants of disease-causing genes.

**Results:**

Sixty-two patients with CFEOM were recruited into this study. Thirty-nine patients were diagnosed with CFEOM1 and 23 with CFEOM3. Forty-nine of the 62 patients with CFEOM carried either *KIF21A* (41/49) or *TUBB3* variants (8/49). Six known missense variants in the *KIF21A* and *TUBB3* genes, and a novel variant (c.3906T > A, p.D1302E) in the *KIF21A* gene were detected. Most patients with CFEOM1 carrying the *KIF21A* mutation displayed isolated CFEOM, whereas patients with CFEOM3 carrying the *TUBB3* mutation had a wide range of clinical manifestations, either CFEOM alone or syndromes. Nystagmus was also present in 12 patients with CFEOM. Furthermore, the MRI findings varied, ranging from attenuation of the extraocular muscles to dysgenesis of the cranial nerves and brain structure.

**Conclusions:**

The novel variants identified in this study will further expand the spectrum of pathogenic variants in CFEOM-related genes. However, no phenotype–genotype correlations were established because of the diversity of the clinical characteristics of these patients.

**Supplementary Information:**

The online version contains supplementary material available at 10.1186/s13023-024-03206-w.

## Introduction

Congenital fibrosis of the extraocular muscles (CFEOM) is a group of rare ocular motor disorders with an estimated prevalence of 1:230,000 [1]. This condition develops after birth and is characterized by nonprogressive ophthalmoplegia and ptosis in one or both eyes. Some patients with CFEOM may have an abnormal head posture, usually with a chin-up position caused by hypotropia and ptosis. Recent advances in genetics and neuroanatomy have proven that CFEOM is caused by maldevelopment of the cranial nerves and their motor neurons [2]. To date, mutations in seven genes, i.e., *KIF21A* (OMIM *608,283), *PHOX2A* (OMIM *602,753), *TUBB3* (OMIM*602,661), *TUBB2B* (OMIM *612,850), *TUBA1A* (OMIM *602,529), *ECEL1* (OMIM *605,896), and *COL25A1* (OMIM *610,004), have been associated with CFEOM [3, 4]. However, with the exception of *TUBB3*, for which a phenotype–genotype correlation has been suggested in some pathogenic variants, no correction between phenotype and genotype has been established for these genes [5–7].

*KIF21A* is located on chromosome 12q12 and is expressed in the brain, spinal cord, and other structures of the nervous system. It encodes kinesin-like motor proteins that interact directly with microtubules in the nervous system and regulate microtubule dynamics. Microtubules are composed of alpha and beta tubulin and play an important role in transporting essential cellular components along axons and dendrites [8]. The *TUBB3* gene is located on chromosome 16q24.3 and is widely expressed in neurons, the brain, the spinal cord, sensory organs, and, to a lower extent, in the testes. This gene encodes a Class III member of beta tubulin that heterodimerizes with alpha tubulin and assembles to form the microtubules that are involved in neurogenesis and axonal guidance and maintenance [7]. Mutations in *KIF21A* and *TUBB3* are suggested to alter microtubule dynamics and the tubulin heterodimer angle, resulting in CFEOM [9]. However, it is not known whether the *TUBB3*-associated CFEOM and *KIF21A*-associated CFEOM variants share a common pathogenic mechanism.

Based on the clinical features and inheritance pattern, CFEOM is subdivided into five subtypes (1 to 5) [3]. CFEOM1 is the most typical and common type of all forms of CFEOM; it is inherited in an autosomal dominant manner with complete penetrance, and is caused by mutations in *KIF21A*, resulting in dysgenesis of the superior division of the third cranial nerve and the motor neurons, leading to atrophy of its innervated superior rectus muscles and the levator palpebrae superioris. Patients with CFEOM1 are unable to raise their eyes above the midline, presenting as hypotropia and ptosis, with their eyes fixed in an infra-ducted position [8, 10].

CFEOM2 is inherited in an autosomal recessive manner and is caused by mutations in the *PHOX2A* gene, which is prominently expressed in developing oculomotor and trochlear motor neurons and is essential for their survival. Mutations in the *PHOX2A* gene would result in dysgenesis of the third and fourth cranial nerves, leading to symptoms similar to those of complete third nerve palsy, presenting with severe ptosis and large exodeviation. In addition, these patients may have small pupils with a poor response to light [11].

CFEOM3 is inherited in an autosomal dominant manner with incomplete penetrance. Its clinical characteristics vary and are less typical than those of CFEOM1 and CFEOM2. Patients with CFEOM3 may have unilateral or bilateral ophthalmoplegia and ptosis, with a variable degree of restriction in the different gaze directions. CFEOM3 is subdivided into three subtypes, i.e., the CFEOM3A–3 C forms, with CFEOM3A being caused by mutations in the *TUBB3* gene, CFEOM3B being caused by mutations in the *KIF21A* gene, and no responsible gene having been identified for CFEOM3C [7, 12, 13]. CFEOM4, also known as Tukel syndrome, has a similar phenotype to that of CFEOM3 and presents with postaxial oligodactyly/oligosyndactyly of the hands [14]. Finally, CFEOM5 is inherited in an autosomal recessive manner and is caused by mutations in the *COL25A1* gene. Similar to CFEOM3, CFEOM5 has variable clinical characteristics. Patients with CFEOM5 may even present with bilateral Duane retraction syndrome [15]. In this study, we aimed to investigate the clinical and genetic characteristics of 62 Han Chinese patients with CFEOM.

## Methods

### Participants

This is a retrospective study. All patients underwent molecular and clinical diagnostics prior to enrollment. ​Patient clinical data and genetic test results were used as part of a retrospective study, subject to informed consent from the patient and approval by the ethics committee of Beijing Children’s Hospital.

Sixty-two Han Chinese patients with CFEOM were enrolled in this study and underwent careful examinations, including slit lamp for the anterior segment, retinoscopy for fundus, refractive status, ocular alignment and motility, and levator palpebrae superior function assessments. The binocular sensory status was evaluated using the Worth 4 dot test or the Bagolini striated glasses at near and distance. The diagnosis of CFEOM is based on the following criteria: (1) onset of nonprogressive restrictive ophthalmoplegia in one eye or both eyes within 6 months of birth and (2) involvement of more than two extraocular muscles in one eye. Magnetic resonance imaging (MRI) was performed using a 3.0-T GE DISCOVERY MR750 scanner with an 8-channel head coil. Genetic testing was performed on all patients and their affected family members under their informed consent, which was obtained from the parents/legal guardians of participants who were younger than 18 years. The study was approved by the ethics committee of Beijing Children’s Hospital and was in accordance with the tenets of the Declaration of Helsinki.

### Genetic testing

Three microliters of peripheral blood samples were collected from all participants and available nuclear family members, and genomic DNA was extracted using a Blood DNA Kit (Roche Biochemical, Inc). Panel-based next-generation sequencing (NGS) was commercially performed by the Mygenomic Biochemical Company (Beijing, China). A DNA library was constructed and sequenced on an Illumina HiSeq X Ten platform (Illumina, San Diego, USA). The amplified DNA was captured using the self-designed GenCap Capture Kit (MyGenostics Inc, Beijing, China), which consists of 235 eye-disease-causing genes selected from the Orphanet (https://www.orpha.net) and OMIM databases (https://www.omim.org/), in which the probe is designed to capture the whole coding and non-coding regions known to be involved in pathogenic variants associated with eye diseases, including CFEOM. The 235 genes included in the GenCap Capture Kit are listed in Supplementary Table 2. Based on methods reported previously, the quality of the reads was controlled by a serious of procedures [16–19]. First, the low-quality (< 80 bp) and duplicated reads were removed from the raw data using the Cutadapt (https://cutadapt.readthedocs.io/en/stable/) and Sentieon (https://www.sentieon.com/) software, respectively. The clean reads (< 150 bp) were aligned to the UCSC hg19 human reference genome and then analyzed using the Genome Analysis Toolkit (GATK) program, to identify single-nucleotide variants (SNVs) and small insertions or deletions (indels). Only variants with a GATK-assigned quality criterion score > 50.0 and a minor allele frequence < 0.01 were considered for downstream analysis. Variants were further annotated using the ANNOVAR software (http://annovar.openbioinformatics.org/en/latest/), which combines multiple public databases, including 1000 Genomes, ESP6500, dbSNP, genomAD, ClinVar, HGMD, and the commercial MyGenostics database. Copy number variants were detected using the CNV kit based on the read-depth algorithm [20]. The pathogenicity of missense mutations was predicted using PolyPhen 2 (http://genetics.bwh.harvard.edu/pph2/), Sorting Intolerant from Tolerant (SIFT, https://sift.bii.a-star.edu.sg/), and Combined Annotation Dependent Depletion (CADD) (https://cadd.gs.washington.edu/). The splicing effects of the variants were assessed using the SpliceAI program [21] and CADD. All potential pathogenic variants identified by NGS were further validated using the Sanger sequencing method. A co-segregation analysis of variants in the family was performed using Sanger sequencing. The primer pairs used in the Sanger method are listed in Supplementary Table 3. The pathogenicity of the variant was interpreted according to the guidelines of the American College of Medical Genetics and Genomics (ACMG) [22]. A 3-dimension (3D) model of the non-synonymous mutant protein was generated and visualized using the PyMOL program (https://pymol.org/) [23].

## Results

### Demographics and clinical features

**Fig. 1:**
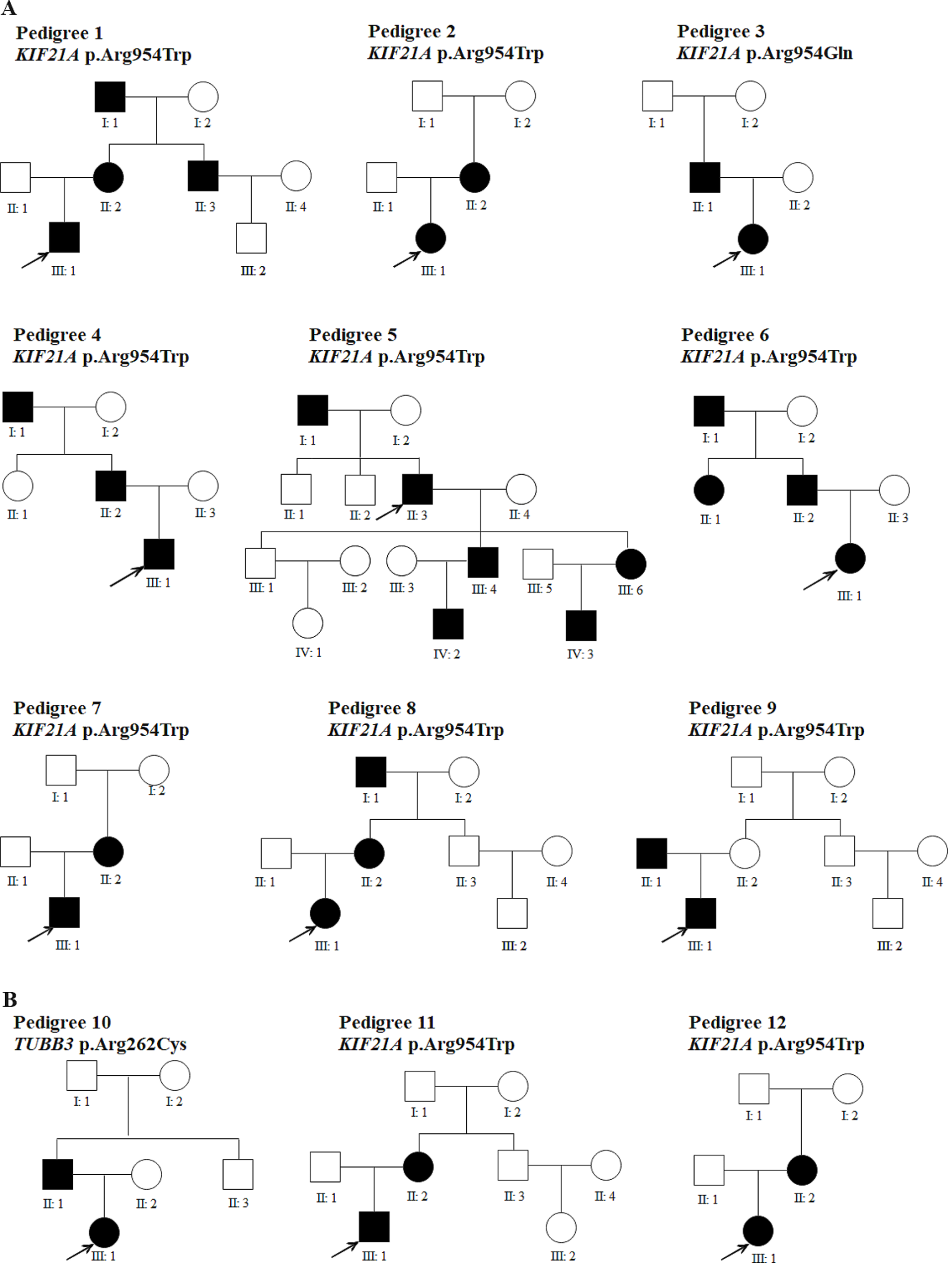
Pedigrees of CFEOM families carrying variants. (**A**) Pedigrees of CFEOM1 families carrying *KIF21A* variants. (**B**) Pedigrees of CFEOM3 families carrying *KIF21A* and *TUBB3* variants

Of the 62 affected individuals, 33 patients were recruited from 12 pedigrees with an autosomal dominant inheritance pattern (Fig. 1; Table 1), whereas 29 patients were sporadic cases (Table 1). Based on their clinical characteristics, 39 patients (62.90%, 39/62) were identified as having CFEOM1 and 23 (37.10%, 23/62) were diagnosed as having CFEOM3. Nine of the 12 pedigrees were identified as being CFEOM1 and three as CFEOM3. Twelve patients with sporadic disease (41.38%, 12/29) were diagnosed with CFEOM1 and 17 (58.62%, 17/29) with CFEOM3. Their anterior and fundus were normal under slit-lamp and retinoscopy examinations. None of the cooperative patients had binocular vision, as evaluated by the Worth 4 dot test or the Bagolini striated glasses. Thirty-two patients (51.61%, 32/62) with CFEOM had a sluggish pupillary response to light, even among patients with CFEOM1. Among the 53 patients who cooperated with undergoing refractive examination, 95 eyes had astigmatism over 0.25D, 50 eyes (52.63%) had rule astigmatism, 29 eyes (30.52%) and against-the-rule astigmatism, and 16 eyes (16.84%) had oblique astigmatism. All patients with CFEOM1 had bilateral blepharoptosis, ophthalmoplegia with both eyes fixed in an infra-ducted position, an inability to move the eyes above the horizontal midline, and a compensatory head position of chin-up. Among the patients with CFEOM1, 30 presented (76.92%, 30/39) with exotropia, four (10.26%, 4/39) with esotropia, and five (12.82%, 4/39) with orthophoria in the horizontal direction. Patients with CFEOM3 had restrictive strabismus in one or both eyes. Six out of 23 patients (26.09%, 6/23) with CFEOM3 presented with monocular ophthalmoplegia, and 17 (73.91%, 17/23) of them exhibited binocular ophthalmoplegia. Their strabismus may appear as a combination of deviations in the horizontal and vertical directions, either exotropia, esotropia, or orthophoria in the horizontal direction, with or without vertical deviations. Seventeen (73.91%, 17/23) of the 23 patients with CFEOM3 had variable degrees of ptosis, and six (26.09%, 6/23) did not exhibit ptosis.

**Table 1:** Clinical and genetic features of patients with CFEOM

No.	*P*/S	patient	Visiting age (years)	G	Genetic findings		BCVA	SE		Ptosis	Ocular alignment		ROM		Nys	SPLR	Non-ocular findings	Brain malformations (MRI)
					cDNA variations	AA change	(*R*/L)	*R*	L		V	H	V	H				
CFEOM1																		
1	P1	I1	55	M	2860 C > T	Arg954Trp	0.3/0.2	-1.5	0.5	B	HoT	XT	+	+	-	+	-	/
2	P1	II2	30	F	2860 C > T	Arg954Trp	0.2/0.9	-1.5	-0.25	B	HoT	XT	+	+	-	+	-	/
3	P1	II3	26	M	2860 C > T	Arg954Trp	0.2/0.3	-1.25	-1.125	B	HoT	XT	+	+	-	+	-	/
4	P1	III1	7	M	2860 C > T	Arg954Trp	0.1/0.1	5.875	5.75	B	HoT	XT	+	+	+	+	-	EOMs; CN3
5	P2	II2	26	F	2860 C > T	Arg954Trp	0.6/0.6	1.25	0.125	B	HoT	XT	+	+	-	-	-	/
6	P2	III1	3	F	2860 C > T	Arg954Trp	/	/	/	B	HoT	Ortho	+	+	+	-	-	EOMs; CN3
7	P3	II1	30	M	2861G > A	Arg954Gln	0.2/0.3	-1.625	-2.375	B	HoT	XT	+	+	-	-	-	/
8	P3	III1	6	F	2861G > A	Arg954Gln	0.2/0.3	3	2.25	B	HoT	XT	+	+	-	-	-	EOMs; CN3
9	P4	I1	58	M	2860 C > T	Arg954Trp	0.2/0.3	-1.25	-1.75	B	HoT	XT	+	+	-	+	-	/
10	P4	II2	30	M	2860 C > T	Arg954Trp	0.6/0.1	0.25	0.375	B	HoT	XT	+	+	-	+	-	/
11	P4	III1	5	M	2860 C > T	Arg954Trp	0.4/0.1	3	3.75	B	HoT	XT	+	+	-	+	-	EOMs; CN3
12	P5	I1	78	M	2860 C > T	Arg954Trp	0.1/0.1	-2.375	-2.625	B	HoT	XT	+	+	-	+	-	/
13	P5	II3	58	M	2860 C > T	Arg954Trp	0.2/0.1	0.25	0.375	B	HoT	ET	+	+	-	+	-	EOMs; CN3
14	P5	III4	36	M	2860 C > T	Arg954Trp	0.3/0.1	-3.375	0.25	B	HoT	XT	+	+	-	+	-	/
15	P5	III6	34	F	2860 C > T	Arg954Trp	0.2/0.3	-2.375	-0.75	B	HoT	XT	+	+	-	+	-	/
16	P5	IV2	11	M	2860 C > T	Arg954Trp	0.1/0.3	0.875	0.5	B	HoT	XT	+	+	-	+	-	/
17	P5	IV3	8	M	2860 C > T	Arg954Trp	0.2/0.3	-0.375	0.25	B	HoT	ET	+	+	-	+	-	/
18	P6	II1	29	F	2860 C > T	Arg954Trp	0.3/0.5	-1.75	-1	B	HoT	XT	+	+	-	+	-	/
19	P6	II2	32	M	2860 C > T	Arg954Trp	0.4/0.3	-3.125	-2.375	B	HoT	XT	+	+	-	+	-	/
20	P6	III1	7	F	2860 C > T	Arg954Trp	0.6/0.6	2.375	2.5	B	HoT	XT	+	+	-	+	-	EOMs; CN3
21	P7	II2	28	F	2860 C > T	Arg954Trp	1.0/0.2	-0.75	-0.5	B	HoT	XT	+	+	-	-	-	/
22	P7	III1	3	M	2860 C > T	Arg954Trp	/	3.875	3.375	B	HoT	Ortho	+	+	-	-	-	EOMs; CN3
23	P8	I1	62	M	2860 C > T	Arg954Trp	0.3/0.2	-0.625	-0.5	B	HoT	XT	+	+	-	+	-	/
24	P8	II2	29	F	2860 C > T	Arg954Trp	0.3/0.4	-4.375	0.25	B	HoT	XT	+	+	-	+	-	/
25	P8	III1	5	F	2860 C > T	Arg954Trp	0.4/0.2	2.75	2.125	B	HoT	XT	+	+	-	+	-	EOMs; CN3
26	P9	II1	32	M	2860 C > T	Arg954Trp	1.0/0.1	-0.25	0.375	B	HoT	XT	+	+	-	+	-	/
27	P9	III1	3	M	2860 C > T	Arg954Trp	/	/	/	B	HoT	Ortho	+	+	-	+	-	EOMs; CN3
28	S1		6	F	2860 C > T	Arg954Trp	0.1/0.3	6.375	-0.125	B	HoT	ET	+	+	+	-	-	/
29	S2		3	F	2860 C > T	Arg954Trp	/	/	/	B	HoT	Ortho	+	+	-	+	-	EOMs; CN3; CN6
30	S3		9	F	2860 C > T	Arg954Trp	0.1/0.2	1.25	3	B	HoT	XT	+	+	+	-	-	/
31	S4		5	F	3906T > A	Asp1302Glu	0.1/0.1	4.875	6.625	B	HoT	XT	+	+	-	-	-	EOMs; CN3
32	S5		4	F	2860 C > T	Arg954Trp	/	2.25	2.375	B	HoT	XT	+	+	+	-	-	EOMs; CN3
33	S6		3	F	2861G > A	Arg954Gln	/	/	/	B	HoT	Ortho	+	+	-	-	-	EOMs; CN3
34	S7		3	F	2861G > A	Arg954Gln	/	3.5	2.875	B	HoT	ET	+	+	-	-	-	/
35	S8		5	M	2861G > A	Arg954Gln	0.3/0.1	0.125	-0.125	B	HoT	XT	+	+	-	-	-	/
36	S9		5	F	2860 C > T	Arg954Trp	0.1/0.2	5.25	5.375	B	HoT	XT	+	+	+	-	-	EOMs
37	S10		4	M	2860 C > T	Arg954Trp	0.3/0.2	5	5.25	B	HoT	XT	+	+	-	-	-	EOMs; CN3
38	S11		5	M	2860 C > T	Arg954Trp	0.2/0.5	0.5	-2	B	HoT	XT	+	+	-	-	-	EOMs; CN3
39	S12		6	M	2861G > A	Arg954Gln	0.2/0.3	2.5	3.25	B	HoT	XT	+	+	-	-	-	EOMs
CFEOM3																		
40	P10	II1	32	M	784 C > T	Arg262Cys	0.4/0.3	1.25	3	B	HoT	XT	+	+	-	+	-	/
41	P10	III1	5	F	784 C > T	Arg262Cys	0.3/0.2	3.75	4.625	B	HoT	XT	+	+	-	+	-	/
42	P11	II2	33	F	2860 C > T	Arg954Trp	0.2/0.5	-1.75	-1	B	HoT	ET	+	-	-	-	-	/
43	P11	III1	8	M	2860 C > T	Arg954Trp	0.1/0.5	2.5	4	B	HoT	ET	+	-	-	-	-	/
44	P12	II2	31	F	2860 C > T	Arg954Trp	0.6/0.2	3	3.75	B	HoT	XT	+	+	-	+	-	/
45	P12	III1	6	F	2860 C > T	Arg954Trp	0.5/0.4	2.5	-0.5	B	HoT	ET	+	+	-	-	-	EOMs; CN3; CN6
46	S13		3	F	-	-	/	2.375	2.875	-	Ortho	XT	+	+(R)	-	-	-	/
47	S14*		2 m	M	-	-	/	/	/	-	HoT(R)	ET	+(R)	+(R)	-	-	-	EOMs
48	S15*		2	F	-	-	/	/	/	R	HoT(R)	XT	+(R)	+(R)	-	-	-	/
49	S16		4	M	1228G > A	Glu410Lys	/	-2.375	-3.625	B	HoT	XT	+	+	+	+	FW, DD, KS, VCP	EOMs; CN3; CN6; CN7; CC; LV
50	S17		3	M	1228G > A	Glu410Lys	/	/	/	B	HoT	XT	+	+	+	+	FW, DD, KS, VCP	EOMs; CN3; CC; OBS
51	S18		3	M	-	-	/	4.25	3.625	L	HoT	ET	+	+(L)	-	+	-	/
52	S19		4	F	-	-	0.1/0.2	5.75	0.25	-	Ortho	ET	+	+	+	-	-	EOMs; CN3
53	S20		5	F	-	-	0.5/0.1	2.5	2.625	B	Ortho	ET	+	+	-	+	-	EOMs; CN6
54	S21		3	M	904G > A	Ala302Thr	/	5.25	7.25	B	HoT	Ortho	+	+	+	+	-	EOMs; CN3; CC
55	S22		6	F	-	-	0.8/0.8	-0.5	-0.625	-	Ortho	Ortho	+	+	-	-	scoliosis	/
56	S23*		30	F	-	-	0.1/1.0	0.75	-0.5	R	HoT(R)	ET	+(R)	+(R)	-	-	-	/
57	S24		6	F	1249G > A	Asp417Asn	0.6/0.1	0.125	2.25	L	HoT	XT	+	+(L)	-	-	-	EOMs; CN3; CN6
58	S25*		3	M	-	-	/	2.375	4.125	-	HoT(L)	XT	+(L)	+(L)	-	-	-	EOMs; CN3
59	S26		5	M	1228G > A	Glu410Lys	/	4.5	4.5	B	HoT	XT	+	+	-	+	DD, KS	EOMs; CN3; CC
60	S27		1	F	1228G > A	Glu410Lys	/	/	/	B	HoT	XT	+(R)	+	+	+	FW, DD, KS	EOMs; CN3
61	S28*		3	M	-	-	/	4.125	3.25	-	HoT(R)	XT	+(R)	+(R)	+	-	-	/
62	S29*		3	M	-	-	/	/	/	R	HoT(R)	XT	+(R)	+(R)	-	-	-	EOMs

Abbreviation: P: Pedigree; S: Sporadic; G: Gender; AA: Amino acid; BCVA: Best corrected visual acuity; SE: Spherical equivalent; ROM: Restriction of ocular movement; Nys: Nystagmus; SPLR: Sluggish pupillary light reflex; R: Right; L: Left; SPLR: Sluggish pupillary light reflex; V: Vertical; H: Horizontal; ET: Esotropia; XT: Exotropia; Ortho: Orthophoria; EOMs: Extraocular muscles; FW: Facial weakness; DD: Developmental delay; KS: Kallmann syndrome; VCP: Vocal cord paralysis; OBS: Olfactory bulbs and sulci; CC: Corpus callosum; LV: Lateral ventricl; /: Not available; *: Monocular ophthalmoplegia

**Fig. 2:**
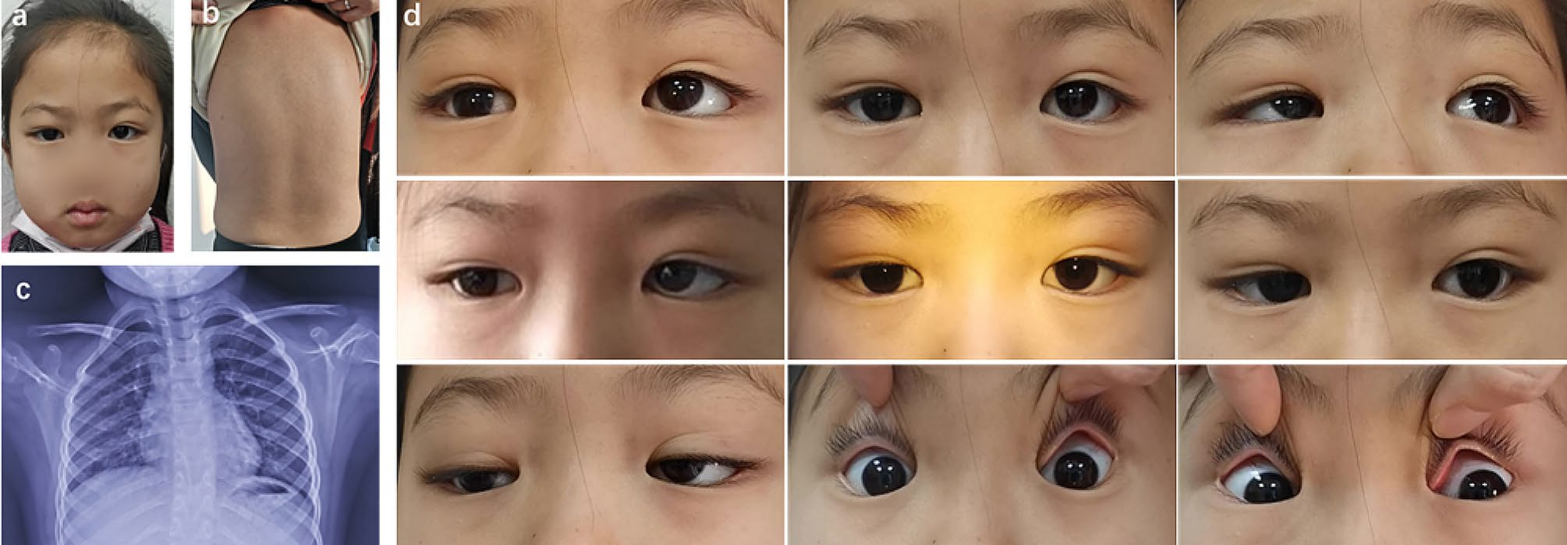
Clinical spectrum and spine X-ray of patient S22 with CFEOM3. (**a**) Facial photograph showing the normal bilateral palpebral fissure widths and orthophoria on the primary eye position. (**b**) Body photograph indicating scoliosis. (**c**) Thoracic X-ray film of scoliosis. (**d**) Nine gaze photographs showing limited eye movements in both the horizontal and vertical directions, which differentiates the condition of the patient from horizontal gaze palsy with progressive scoliosis

Nystagmus was detected in only 12 patients. Horizontal jerk nystagmus was found in six patients with CFEOM1 and four patients with CFEOM3 (S17, S19, S27, S28). Upbeat and multidirectional nystagmus was respectively identified from each of two patients with CFEOM3 (S21, and S16). In addition, other symptoms were observed in patients with CFEOM3, including facial weakness in three patients (S16, S17, S27), developmental delay in four patients (S16, S17, S26, S27), Kallmann syndrome (hypogonadotropic hypogonadism with anosmia) in four patients (S16, S17, S26, S27), vocal cord paralysis in two patients (S16, S17), and scoliosis in one patient (S22) (Table 1; Fig. 2).

Twenty-nine patients underwent MRI. The MRI findings included attenuation of the extraocular muscles in all patients, hypoplasia of oculomotor nerve in 24 (82.76%, 24/29) patients (15 CFEOM1, 9 CFEOM3), dysgenesis or agenesis of the sixth cranial nerve in five patients (S2, S16, S20, S24, and P12-III1), dysgenesis of the seventh cranial nerve in a patient with CFEOM3 (S16), hypoplasia of the corpus callosum in four patients with CFEOM3 (S16, S17, S21 and S26), aplasia of the olfactory bulb and sulcus in a patient with CFEOM3 (S17), and temporal horn enlargement of the lateral ventricle in a patient with CFEOM3 (S16). The demographics and detailed clinical characteristics of the patients with CFEOM are listed in Table 2 (additional information is provided in Supplementary Table 1).

**Table 2:** Demographics and clinical features of CFEOM patients

		CFEOM1	CFEOM3	
			Unilateral	Bilateral
No. of patients	39		6	17
sex				
Male	21		3	8
Female	18		3	9
Pedigrees	9		0	3
Sporadic cases	12		6	11
BCVA (mean ± SD)	0.29 ± 0.21		0.55 ± 0.64	0.37 ± 0.22
SE (mean ± SD)	0.92 ± 2.61		2.20 ± 2.58	2.35 ± 1.89
***Primary horizontal position of eyes***				
Orthophoria		5	0	2
Exotropia		30	4	9
Esotropia		4	2	6
***Primary vertical position of eyes***				
Orthophoria		0	0	3
hypotropia		37	6	14
***Restriction of eye movements***				
Vertical		37	6	17
Horizontal		37	6	15
Blepharoptosis		37	4	13
Nystagmus		6	1	5
SPLR		22	1	9
MRI (performed)		17	2	10
***Abnormalities in MRI***				
EOMs		17	2	10
CN3		15	1	8
CN6		1	0	4
CN7		0	0	1
CC		0	0	4
OBS		0	0	1
LV		0	0	1
***Non-ocular findings***				
facial weakness		0	0	3
developmental delay		0	0	4
Kallmann syndrome		0	0	4
vocal cord paralysis		0	0	2
scoliosis		0	0	1

Abbreviation: BCVA: Best corrected visual acuity; SE: Spherical equivalent; SPLR: Sluggish pupillary light reflex; EOMs: Extraocular muscles; CN3: oculomotor nerve; CN6: abducens nerve; CN7: facial nerve; CC: Corpus callosum; OBS: Olfactory bulbs and sulci; LV: lateral ventricle

### Genetic analysis

**Fig. 3:**
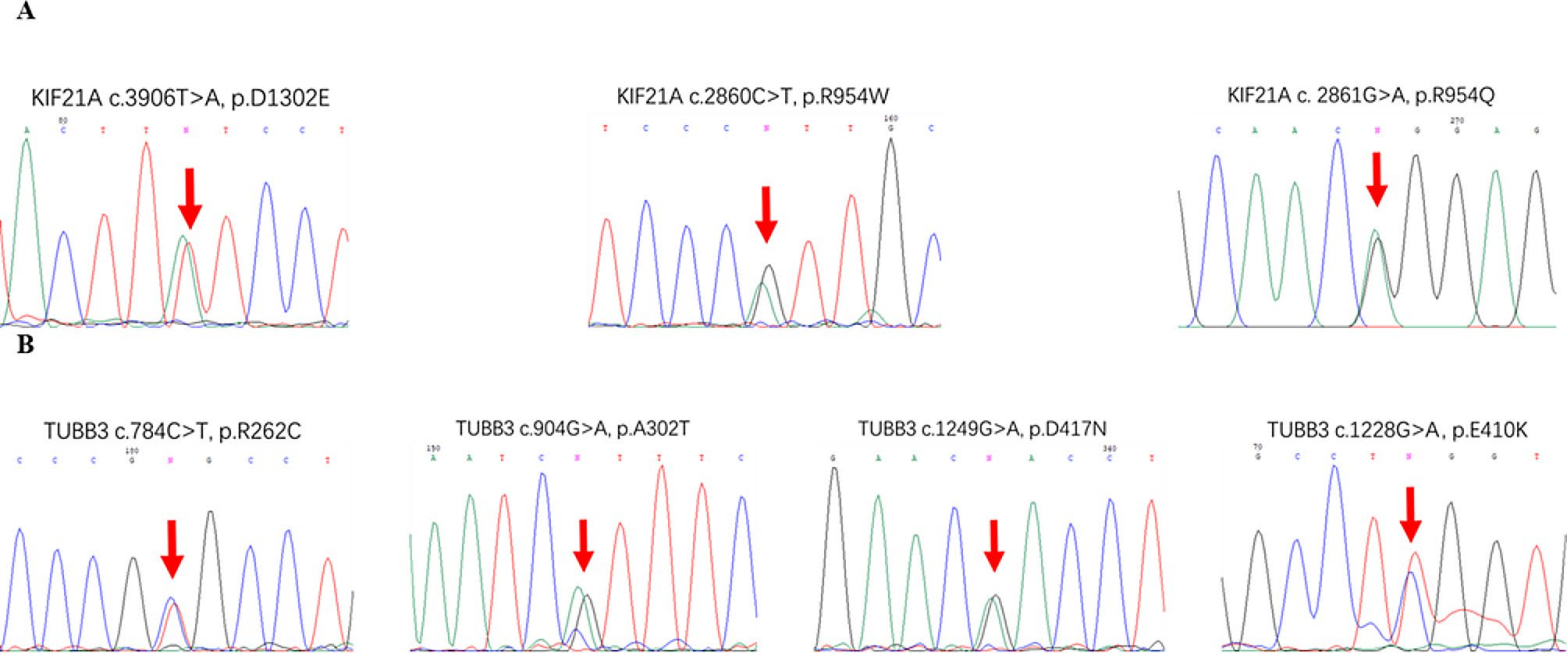
Genomic sequence chromatograms of patients with CFEOM carrying *KIF21A* and *TUBB3* variants. (**A**) Three heterozygous variants of the *KIF21A* gene (red arrows). (**B**) Four heterozygous variants of the *TUBB3* gene (red arrows)

**Fig. 4:**
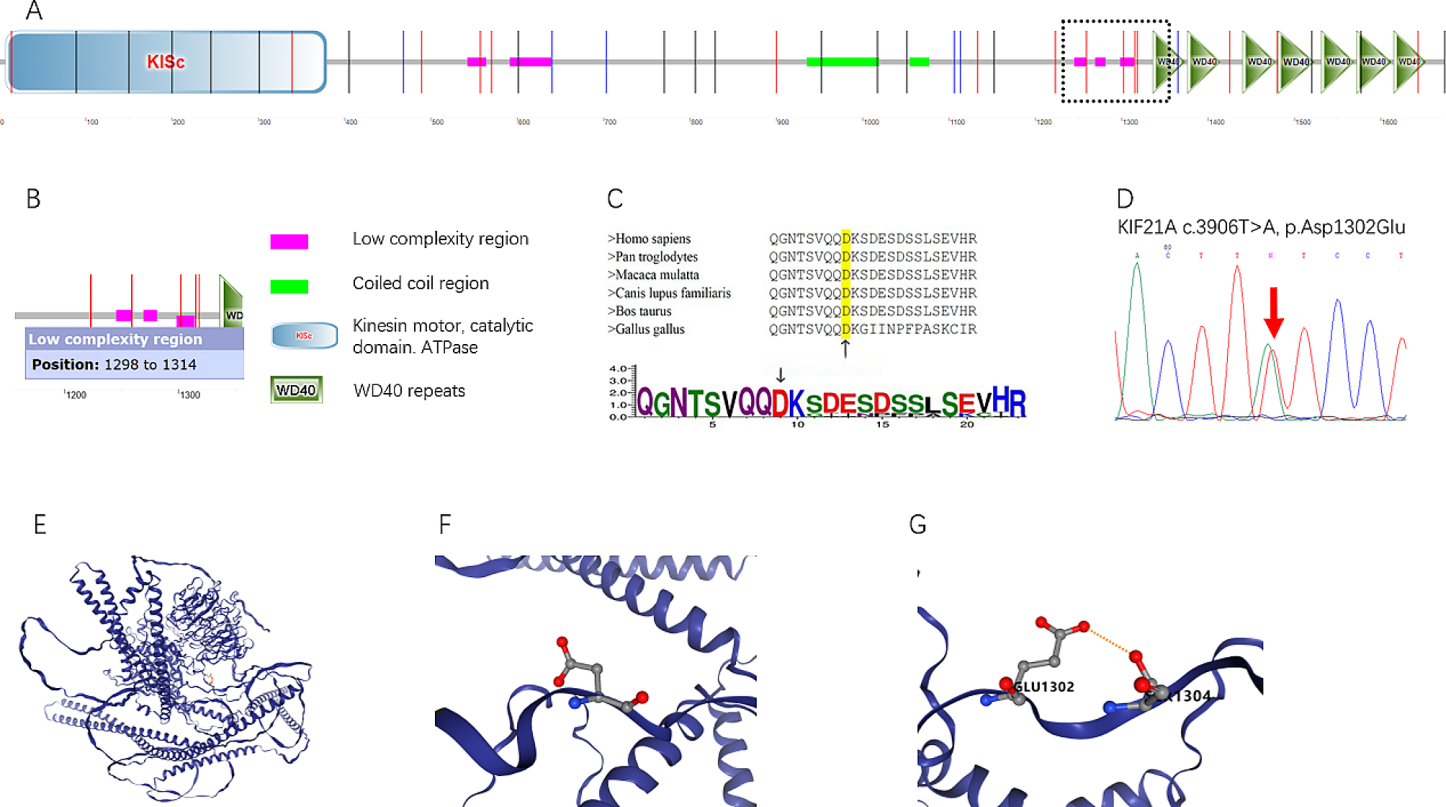
Genetic feature of variant c.3906T > A(p.Asp1302Glu) of *KIF21A*. (**A**) Domain structure of the *KIF21A* protein. (**B**) The D1302 residue is located in the low-complexity region of *KIF21A*. (**C**) Multiple sequence alignment demonstrates high conservation of the p.Asp1302 residue (marked with black arrows). (**D**) Genomic sequence chromatograms of the c.3906T > A(p.Asp1302Glu) variant. **E, F.** Schematic representation of the structure and spatial distribution of the D1302 residue of the KIF21A protein. **G.** Interaction force of the Asp1302Glu substitution with the surrounding amino acid residues

Forty-nine of the 62 patients carried variants in the *KIF21A* and *TUBB3* genes, with 41 patients carrying a missense variant of the *KIF21A* gene and eight patients carrying a missense variant of the *TUBB3* gene. Only three types of missense variants, i.e., c.3906T > A(p.Asp1302Glu), c.2860 C > T(p.Arg954Trp), and c.2861G > A(p.Arg954Gln), were identified in the *KIF21A* gene, and four types of variants, i.e., c.784 C > T(p.Arg262Cys), c.904G > A(p.Ala302Thr), c.1249G > A(p.Asp417Asn), and c.1228G > A(p.Glu410Lys), were detected in the *TUBB3* gene. With the exception of c.3906T > A(p.Asp1302Glu) in *KIF21A*, the remaining variants in *KIF21A* and *TUBB3* had been reported previously and were present in more than one affected individual, especially for c.2860 C > T(p.Arg954Trp) and c.2861G > A(p.Arg954Gln) in *KIF21A* (Fig. 3). The c.3906T > A(p.Asp1302Glu) variant of *KIF21A* is regarded as a pathogenic variant because the wild-type aspartic acid (Asp or D) amino acid at codon 1302 is highly conserved among *Homo sapiens*, *Pan troglodytes*, *Macaca mulatta*, *Canis lupus familiaris*, *Bos taurus*, and *Gallus gallus* (Fig. 4). Substitution of aspartic acid (D) with glutamic acid (Glu or E) would destroy the stability of the protein, as predicted by the online SIFT, Polyphen-2, MutationTaster, and CADD programs (Table 3) and further confirmed by the 3-D model that was constructed using Dynamut2 (http://biosig.unimelb.edu.au/dynamut2/), in which the mutant type gained an extra hydrogen bond with the serine (S) amino acid at codon 1304 and resulted in a change in the free energy of the structure (Fig. 4; Table 4).

**Table 3:** Pathogenicity prediction scores for the c.3906T > A(p.Asp1302Glu) substitution

Gene	variant	SIFT	Poly-phen2	MutationTaster	CADD
*KIF21A*	c.3906T > A p.Asp1302Glu	0.882 Tolerable	0.99 Probably damaging	0.989 Disease causing	16.59 Tolerable

**Table 4:** Stability analysis of c.3906T > A(p.Asp1302Glu) using different servers and software

	DynaMut	ENCoM	mCSM	SDM	DUET
ΔΔG (kcal/mol)	-0.118	-0.019	-0.435	0.7	0.037
Outcome	Destabilizing	Destabilizing	Destabilizing	Stabilizing	Stabilizing

### Genotype–phenotype correlations

Nine pedigrees (P1–9) with CFEOM1 carried either the c.2860 C > T(p.Arg954Trp) or c.2861G > A(p.Arg954Gln) variant of *KIF21A*, and two pedigrees (P11–12) with CFEOM3 carried the c.2860 C > T(p.Arg954Trp) variant of *KIF21A*. One pedigree (P10) with CFEOM3 carried the c.784 C > T(p.Arg262Cys) pathogenic variant of *TUBB3* (Fig. 1).

Eleven out of the 12 sporadic patients with CFEOM1 carried either the c.2860 C > T(p.Arg954Trp) or c.2861G > A(p.Arg954Gln) variant of *KIF21A*, with one of them (S4) carrying the novel c.3906T > A(p.Asp1302Glu) missense variant of *KIF21A*. Six of the 11 sporadic patients with bilateral ophthalmoplegia who were diagnosed as having CFEOM3 carried the *TUBB3* variants of c.904G > A(p.Ala302Thr), c.1249G > A(p.Asp417Asn), and c.1228G > A(p.Glu410Lys), whereas five of them did not carry either *KIF21A* or *TUBB3* mutations.(Table 1).

Patients with the c.3906T > A(p.Asp1302Glu) and c.2861G > A(p.Arg954Gln) variants in *KIF21A* presented with a typical feature of CFEOM1, whereas patients with the c.2860 C > T(p.Arg954Trp) variant presented with either CFEOM1 or CFEOM3. The clinical characteristics varied among patients with *TUBB3* mutations. CFEOM without systemic disorders was present in patients with the c.784 C > T(p.Arg262Cys), c.904G > A(p.Ala302Thr), and c.1249G > A(p.Asp417Asn) variants of *TUBB3*, whereas a complicated systemic disorder was observed in patients with the c.1228G > A(p.Glu410Lys) variant of *TUBB3*, including developmental delay and Kallmann syndrome in all four patients (S16, S17, S26, and S27), facial weakness in three patients (S16, S17, and S27), and vocal cord paralysis in two patients (S16 and S17) (Table 1).

Horizontal jerk nystagmus was present in six patients with CFEOM1 carrying the c.2860 C > T(p.Arg954Trp) variant of *KIF21A*, and four patients with CFEOM3 had the c.904G > A(p.Ala302Thr) and c.1228G > A(p.Glu410Lys) variants of *TUBB3*. Upbeat nystagmus was found in a patient (S21) with the c.904G > A(p.Ala302Thr) variant of *TUBB3*, and multidirectional nystagmus was identified only in one patient (S16) with the c.1228G > A(p.Glu410Lys) variant of *TUBB3*.

**Fig. 5:**
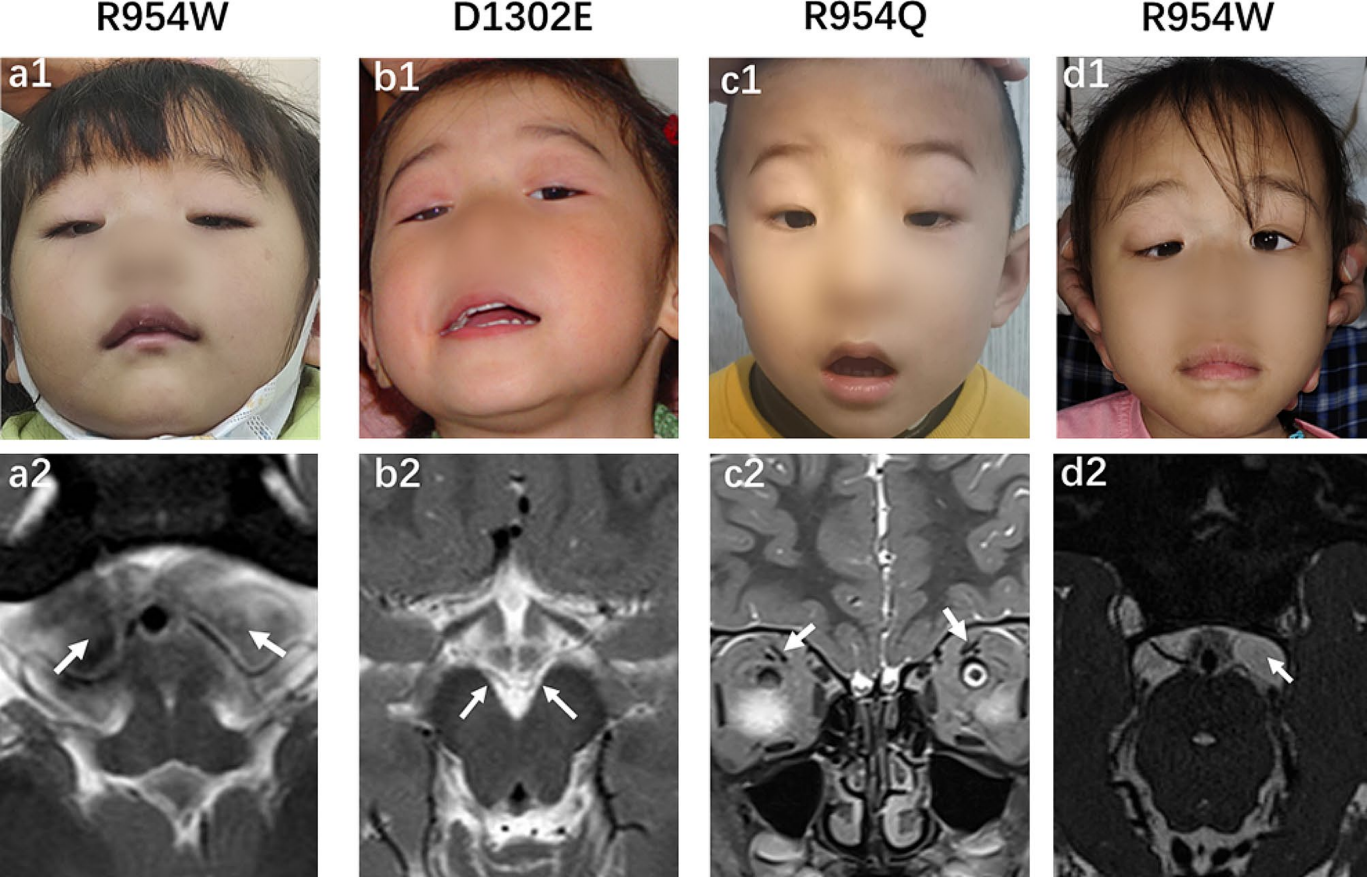
Ocular and MRI findings of patients with CFEOM1 (**a1–c2)** and CFEOM3 (**d1–d2**) carrying *KIF21A* variants. **a1, a2.** Patient S2, harboring the Arg954Trp variant, displayed bilateral ptosis and absence of CN6 on MRI. **b1,b2.** Patient S4, harboring the Asp1302Glu variant, showed bilateral ptosis and hypoplasia of the bilateral CN3 on MRI. **c1,c2.** Patient S6, harboring the Arg954Gln variant, exhibited bilateral ptosis and hypoplasia of the bilateral levator palpebrae superioris-superior rectus on MRI. **d1, d2.** Patient P12-III1, with CFEOM3, harbored the Arg954Trp variant and showed bilateral ptosis and absence of left CN6 on MRI

**Fig. 6:**
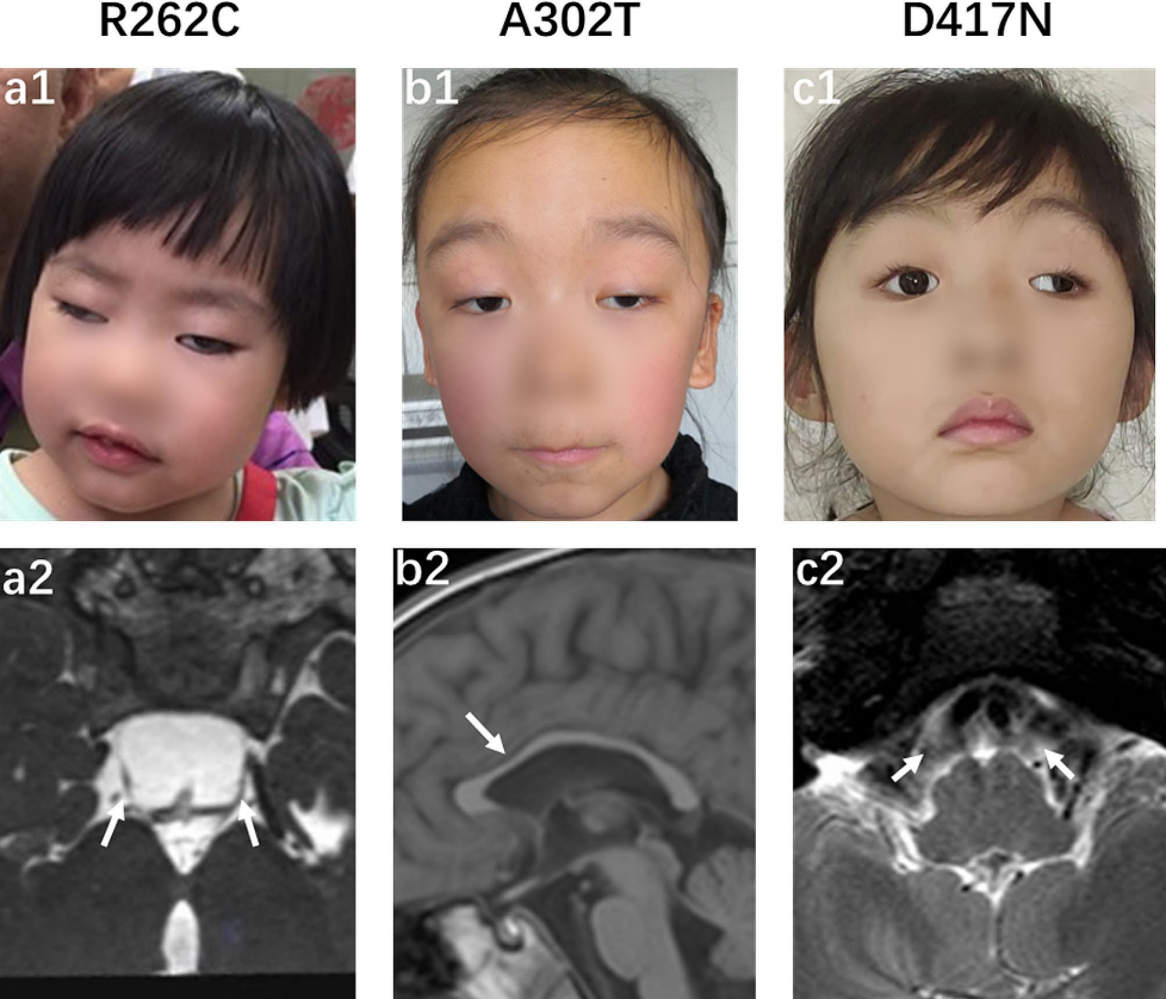
Ocular and MRI findings of patients with CFEOM3 carrying *TUBB3* variants. **a1, a2** Patient P10-III1, harboring the Arg262Cys variant, displayed bilateral ptosis and hypoplasia of CN3 on MRI. **b1, b2** Patient S21, harboring the Ala302Thr variant, showed bilateral ptosis and hypoplasia of the corpus callosum on MRI. **c1, c2** Patient S24, harboring the Asp417Asn variant, exhibited left ptosis and absence of bilateral CN6 on MRI

**Fig. 7:**
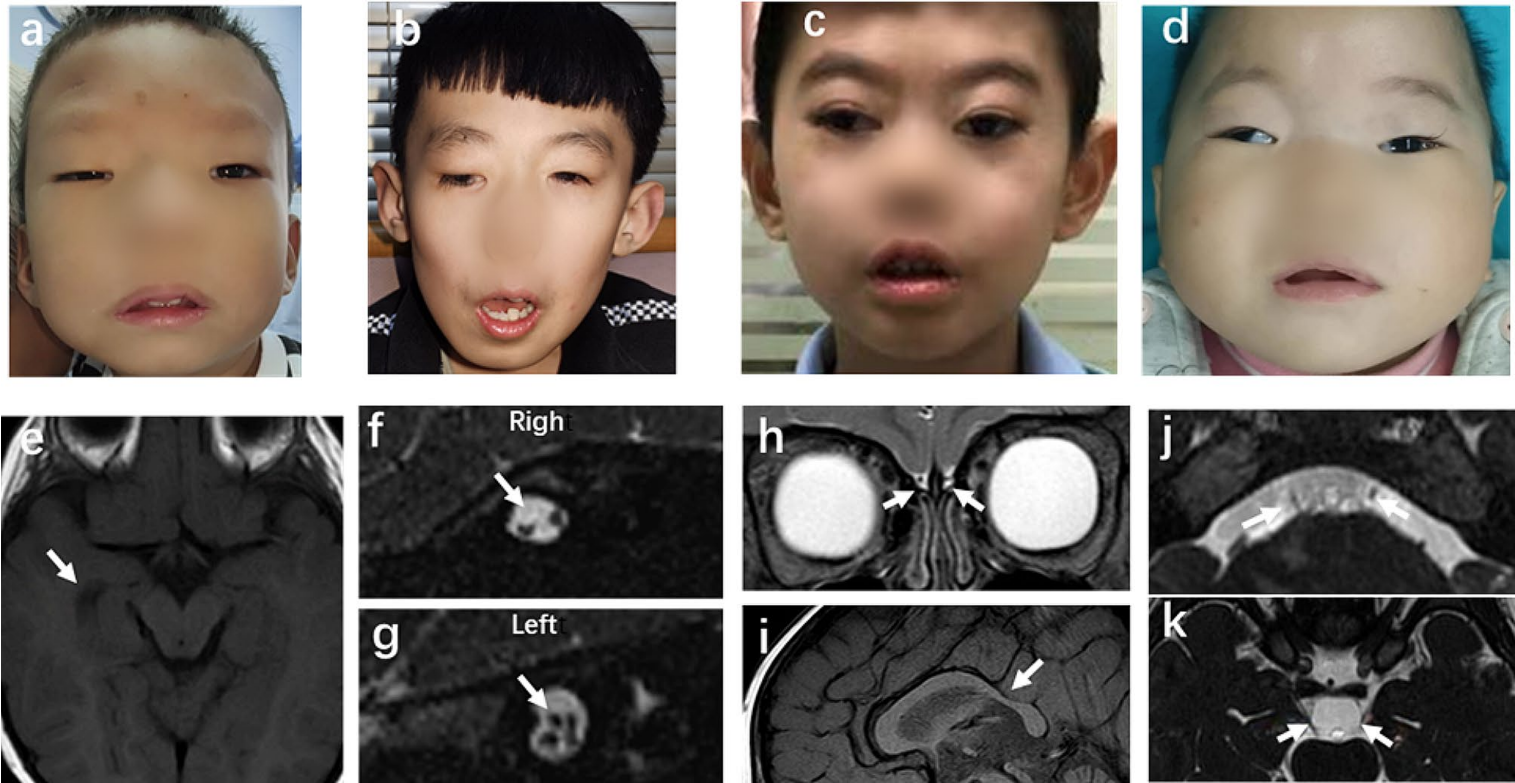
Ocular and MRI findings of four patients with the *TUBB3* Glu410Lys variant. **a–d**. Facial photographs of patients S16 (**a**), S17 (**b**), S26 (**c**), and S27 (**d**), showing bilateral blepharoptosis and facial weakness in patients S16, S17, and S27 (patient S26 had undergone correction of blepharoptosis). **e.** Enlargement of the lateral ventricle on its temporal horn in patient S16. **f, g.** Absence of the right CN7 in patient S16. **h.i.** Hypoplasia of the corpus callosum and the bilateral olfactory bulbs and sulci in patient S17. **j.** Hypoplasia of the bilateral CN6 in patient S26. **k.** Hypoplasia of the bilateral CN3 in patient S27

The S2 and P12-III1 patients, who carried with the same variant of c.2860 C > T(p.Arg954Trp), were found to have aplasia of the sixth cranial nerve on MRI, although they had different types of CFEOM (S2, CFEOM1; and P12-III1, CFEOM3) (Fig. 5; Table 1). Unlike patients with the *KIF21A* mutation, those with the *TUBB3* mutation exhibited more complex and varied findings on MRI (Fig. 6), even though they carried the same variant. Three patients with the Glu410Lys variant had hypoplasia of the corpus callosum and one had an enlarged temporal horn of the lateral ventricle (Fig. 7). Moreover, among the patients carrying the Glu410Lys variant, patient S16 exhibited hypoplasia of the third, six, and seventh cranial nerves and corpus callosum, whereas patient S35 only had hypoplasia of the third cranial nerve (Fig. 7). Because of the complex clinical findings observed in our patients, no correlations between genotype and phenotype were established.

## Discussion

In this study, we evaluated the clinical characteristics of 62 patients with CFEOM and identified six previously reported missense variants in the *KIF21A* and *TUBB3* genes and a novel variant in the *KIF21A* gene in a patient with sporadic CFEOM1. We also found that patients with *TUBB3* variants had more complex and variable clinical features that may be involved in multiple systemic disorders, in contrast to patients with *KIF21A* variants, who had few systemic disorders and only restrictive strabismus.

The KIF21A-encoded protein consists of an amino terminal motor domain, three central coiled-coil stalk domains, and a carboxy-terminal tail domain containing seven WD40 repeats. As a member of the motor kinesin family, KIF21A plays an important role in microtubule-dependent transport from the cell body to the developing growth cone and acts as an inhibitor of microtubule dynamics through interactions with microtubule-associated proteins [8, 24]. To date, at least 15 mutations have been identified in the *KIF21A* gene, most of which are missense mutations and cluster in the second coiled-coil and the motor domains, thus interfering with the function of the protein [12, 25–31]. The novel c.3906T > A(p.Asp1302Glu) variant identified here was located in the low-complexity regions (LCRs), downstream of the third coiled-coil domain. LCRs have been regarded as being involved in numerous aspects of cell biology, including transcriptional regulation, replication, genome stability, and protein–protein interactions [32]. Mutations in LCRs can lead to various human neurological disorders, such as frontotemporal dementia and Alzheimer’s disease [32, 33]. Because aspartate (Asp, D) has a shorter side chain compared with glutamate (Glu, E), it may establish a more stable connection with amino acids other than glutamate when constituting an activity center in the protein. A well-known example of this feature is that aspartate is commonly involved in the formation of protein activation centers via the Asp-His-Ser catalytic triad. Substitution of aspartate (Asp, D) at codon 1304 with glutamate (Glu, E) would destroy the stability of the protein and reduce its activity, which would be predicted to decrease its role as an inhibitor of microtubule dynamics and lead to the stalling of axon guidance [34, 35].

Interestingly, we also found that two patients with the Arg954Trp variant exhibited hypoplasia not only in the third, but also in the six cranial nerve on MRI. Previous studies have shown that hypoplasia of the third and sixth cranial nerves can occur simultaneously in patients with CFEOM [10]. However, specific CFEOM types and their associated genotypes were not mentioned. Definitely, we lack sufficient evidence to support the contention that the hotspot mutation of Arg954Trp is responsible for the hypoplasia of abducens. However, in a recent case report, the syndromic CFEOM phenotype caused by the c.2015T > C(p.Leu672Pro) variant of the *KIF21A* gene illustrates that *KIF21A* mutation may result in multiple damage to the nerve system, rather than only involvement in the ocular motor nerve [12].

The *TUBB3* gene encodes a neuron-specific Class III β-tubulin isotype, which plays a critical role in neuronal migration and axonal guidance [36, 37]. Heterozygous missense mutations in *TUBB3* may cause two distinct phenotypes, isolated or syndromic CFEOM3 or malformations of cortical development, and occasionally both of these conditions [5, 7, 38]. The possible underlying mechanism is that amino acid substitutions in *TUBB3* may disturb the equilibrium of microtubule dynamics and the interaction of microtubules with kinesin motors [7, 39, 40]. In our cohort, patients with the Arg262Cys, Ala302Thr, and Asp417Asn variants of *TUBB3* exhibited isolated CFEOM3 phenotypes. Unlike that reported previously, our patient with the Asp417Asn variant did not have atrophy of the intrinsic foot muscles and an elevated arch [7]. In agreement with the previous reports of the severe clinical findings commonly detected in isolated patients with CFEOM3 with Arg262Cys, one of our patients (P10-III1) with the Arg262Cys variant had a large exodeviation angle of more than 90 PD at the horizontal direction and extremely tight extraocular muscles. Although she has undergone three operations to correct her large exodeviation, she still exhibits recurrent exotropia. Instead of a *de novo* mutation, she inherited the Arg262Cys variant from her affected father. In addition, all four patients with sporadic CFEOM3 carrying the Glu410Lys variant displayed complex clinical manifestations, including facial weakness, developmental delays, and additional systemic disorders, such as microphallus and cryptorchidism. In addition, two patients with the Glu410Lys and Asp417Asn variants had hypoplasia of the third and sixth cranial nerves. In a previous report, nystagmus was present in patients with *TUBB3* mutations [5]; however, in our patients, nystagmus was present not only in patients with *TUBB3* mutations, but also in patients with *KIF21A* mutations. At one point, it was suggested that nystagmus might be related to reduced visual acuity or sensory deficits. However, we suggest that nystagmus would be associated with dysgenesis of the ocular motor control system caused by underdevelopment of the central nervous system.

## Conclusions

We have identified a novel variant in the *KIF21A* gene that will extend the mutation spectrum of *KIF21A* and supply some of the novel phenotypes induced by the *KIF21A* and *TUBB3* mutations. In addition, we described a large cohort of Chinese patients with CFEOM with detailed phenotypes and genotypes.

### Electronic supplementary material

Below is the link to the electronic supplementary material.

Supplementary Material 1

Supplementary Material 2

## Data Availability

The original contributions presented in the study are included in the Supplementary Material, part in https://www.biosino.org/download/node/data/public/OED908528, further inquiries can be directed to the corresponding author.

## References

[1] Reck AC, Manners R, Hatchwell E. Phenotypic heterogeneity may occur in congenital fibrosis of the extraocular muscles. Br J Ophthalmol. 1998;82(6):676–9. 9797671 10.1136/bjo.82.6.676 PMC1722617

[2] Whitman MC, Engle EC. Ocular congenital cranial dysinnervation disorders (CCDDs): insights into axon growth and guidance. Hum Mol Genet. 2017;26(R1). 10.1093/hmg/ddx168. R37-37R44. 10.1093/hmg/ddx168 PMC5886468 28459979

[3] Whitman MC, Jurgens JA, Hunter DG, Engle EC. Congenital fibrosis of the extraocular muscles: overview. 2021. Seattle (WA). 20301522

[4] Khan AO, Shaheen R, Alkuraya FS. The ECEL1-related strabismus phenotype is consistent with congenital cranial dysinnervation disorder. J AAPOS. 2014;18(4):362–7. 10.1016/j.jaapos.2014.03.005 25173900 10.1016/j.jaapos.2014.03.005

[5] Whitman MC, Andrews C, Chan WM, Tischfield MA, Stasheff SF, Brancati F, et al. Two unique TUBB3 mutations cause both CFEOM3 and malformations of cortical development. Am J Med Genet A. 2016;170A(2):297–305. 10.1002/ajmg.a.37362 26639658 10.1002/ajmg.a.37362 PMC4770801

[6] Chew S, Balasubramanian R, Chan WM, Kang PB, Andrews C, Webb BD, et al. A novel syndrome caused by the E410K amino acid substitution in the neuronal β-tubulin isotype 3. Brain. 2013;136(Pt 2):522–35. 10.1093/brain/aws345 23378218 10.1093/brain/aws345 PMC3572929

[7] Tischfield MA, Baris HN, Wu C, Rudolph G, Van Maldergem L, He W, et al. Human TUBB3 mutations perturb microtubule dynamics, kinesin interactions, and axon guidance. Cell. 2010;140(1):74–87. 10.1016/j.cell.2009.12.011 20074521 10.1016/j.cell.2009.12.011 PMC3164117

[8] Yamada K, Andrews C, Chan WM, McKeown CA, Magli A, de Berardinis T, et al. Heterozygous mutations of the kinesin KIF21A in congenital fibrosis of the extraocular muscles type 1 (CFEOM1). Nat Genet. 2003;35(4):318–21. 10.1038/ng1261 14595441 10.1038/ng1261

[9] Puri D, Barry BJ, Engle EC. TUBB3 and KIF21A in neurodevelopment and disease. Front Neurosci. 2023;17:1226181. 10.3389/fnins.2023.1226181 37600020 10.3389/fnins.2023.1226181 PMC10436312

[10] Demer JL, Clark RA, Engle EC. Magnetic resonance imaging evidence for widespread orbital dysinnervation in congenital fibrosis of extraocular muscles due to mutations in KIF21A. Invest Ophthalmol Vis Sci. 2005;46(2):530–9. 10.1167/iovs.04-1125 15671279 10.1167/iovs.04-1125

[11] Nakano M, Yamada K, Fain J, Sener EC, Selleck CJ, Awad AH, et al. Homozygous mutations in ARIX(PHOX2A) result in congenital fibrosis of the extraocular muscles type 2. Nat Genet. 2001;29(3):315–20. 10.1038/ng744 11600883 10.1038/ng744

[12] Soliani L, Spagnoli C, Salerno GG, Mehine M, Rizzi S, Frattini D, et al. A Novel De Novo KIF21A variant in a patient with congenital fibrosis of the extraocular muscles with a syndromic CFEOM phenotype. J Neuroophthalmol. 2021;41(1):e85–8588. 10.1097/WNO.0000000000000921 32141982 10.1097/WNO.0000000000000921

[13] Aubourg P, Krahn M, Bernard R, Nguyen K, Forzano O, Boccaccio I, et al. Assignment of a new congenital fibrosis of extraocular muscles type 3 (CFEOM3) locus, FEOM4, based on a balanced translocation t(2;13) (q37.3;q12.11) and identification of candidate genes. J Med Genet. 2005;42(3):253–9. 10.1136/jmg.2004.021899 15744040 10.1136/jmg.2004.021899 PMC1736008

[14] Tukel T, Uzumcu A, Gezer A, Kayserili H, Yuksel-Apak M, Uyguner O, et al. A new syndrome, congenital extraocular muscle fibrosis with ulnar hand anomalies, maps to chromosome 21qter. J Med Genet. 2005;42(5):408–15. 10.1136/jmg.2004.026138 15863670 10.1136/jmg.2004.026138 PMC1736053

[15] Shinwari JM, Khan A, Awad S, Shinwari Z, Alaiya A, Alanazi M, et al. Recessive mutations in COL25A1 are a cause of congenital cranial dysinnervation disorder. Am J Hum Genet. 2015;96(1):147–52. 10.1016/j.ajhg.2014.11.006 25500261 10.1016/j.ajhg.2014.11.006 PMC4289688

[16] Han P, Wei G, Cai K, Xiang X, Deng WP, Li YB, et al. Identification and functional characterization of mutations in LPL gene causing severe hypertriglyceridaemia and acute pancreatitis. J Cell Mol Med. 2020;24(2):1286–99. 10.1111/jcmm.14768 31901151 10.1111/jcmm.14768 PMC6991700

[17] Zhang R, Chen S, Han P, Chen F, Kuang S, Meng Z, et al. Whole exome sequencing identified a homozygous novel variant in CEP290 gene causes Meckel syndrome. J Cell Mol Med. 2020;24(2):1906–16. 10.1111/jcmm.14887 31840411 10.1111/jcmm.14887 PMC6991682

[18] Dai Y, Liang S, Dong X, Zhao Y, Ren H, Guan Y, et al. Whole exome sequencing identified a novel DAG1 mutation in a patient with rare, mild and late age of onset muscular dystrophy-dystroglycanopathy. J Cell Mol Med. 2019;23(2):811–8. 10.1111/jcmm.13979 30450679 10.1111/jcmm.13979 PMC6349151

[19] Zheng Y, Xu J, Liang S, Lin D, Banerjee S. Whole exome sequencing identified a Novel heterozygous mutation in HMBS gene in a Chinese patient with acute intermittent porphyria with rare type of mild anemia. Front Genet. 2018;9:129. 10.3389/fgene.2018.00129 29731767 10.3389/fgene.2018.00129 PMC5920022

[20] Talevich E, Shain AH, Botton T, Bastian BC, CNVkit. Genome-wide copy number detection and visualization from targeted DNA sequencing. PLoS Comput Biol. 2016;12(4):e1004873. 10.1371/journal.pcbi.1004873 27100738 10.1371/journal.pcbi.1004873 PMC4839673

[21] Jaganathan K, Kyriazopoulou Panagiotopoulou S, McRae JF, Darbandi SF, Knowles D, Li YI, et al. Predicting splicing from primary sequence with deep learning. Cell. 2019;176(3):535–e4824. 10.1016/j.cell.2018.12.015 30661751 10.1016/j.cell.2018.12.015

[22] Richards S, Aziz N, Bale S, Bick D, Das S, Gastier-Foster J, et al. Standards and guidelines for the interpretation of sequence variants: a joint consensus recommendation of the American College of Medical Genetics and Genomics and the Association for Molecular Pathology. Genet Med. 2015;17(5):405–24. 10.1038/gim.2015.30 25741868 10.1038/gim.2015.30 PMC4544753

[23] Rigsby RE, Parker AB. Using the PyMOL application to reinforce visual understanding of protein structure. Biochem Mol Biol Educ. 2016;44(5):433–7. 10.1002/bmb.20966 27241834 10.1002/bmb.20966

[24] van der Vaart B, van Riel WE, Doodhi H, et al. CFEOM1-associated kinesin KIF21A is a cortical microtubule growth inhibitor. Dev Cell. 2013;27(2):145–60. 24120883 10.1016/j.devcel.2013.09.010

[25] Xia CR, Shi LH, Nan J, Hao YZ, Jia Y. Identification of a novel KIF21A gene mutation in a Chinese family with congenital fibrosis of the extraocular muscles. Zhonghua Yan Ke Za Zhi. 2022;58(3):213–4. 35280030 10.3760/cma.j.cn112142-20210915-00424

[26] Ali Z, Xing C, Anwar D, et al. A novel de novo KIF21A mutation in a patient with congenital fibrosis of the extraocular muscles and Möbius syndrome. Mol Vis. 2014;20:368–75. 24715754 PMC3976685

[27] Wang P, Li S, Xiao X, Guo X, Zhang Q. KIF21A novel deletion and recurrent mutation in patients with congenital fibrosis of the extraocular muscles-1. Int J Mol Med. 2011;28(6):973–5. 21805025 10.3892/ijmm.2011.759

[28] Lu S, Zhao C, Zhao K, Li N, Larsson C. Novel and recurrent KIF21A mutations in congenital fibrosis of the extraocular muscles type 1 and 3. Arch Ophthalmol. 2008;126(3):388–94. 18332320 10.1001/archopht.126.3.388

[29] Chan WM, Andrews C, Dragan L, et al. Three novel mutations in KIF21A highlight the importance of the third coiled-coil stalk domain in the etiology of CFEOM1. BMC Genet. 2007;8:26. 17511870 10.1186/1471-2156-8-26 PMC1888713

[30] Yamada K, Hunter DG, Andrews C, Engle EC. A novel KIF21A mutation in a patient with congenital fibrosis of the extraocular muscles and Marcus Gunn jaw-winking phenomenon. Arch Ophthalmol. 2005;123(9):1254–9. 16157808 10.1001/archopht.123.9.1254

[31] Yamada K, Chan WM, Andrews C, et al. Identification of KIF21A mutations as a rare cause of congenital fibrosis of the extraocular muscles type 3 (CFEOM3). Invest Ophthalmol Vis Sci. 2004;45(7):2218–23. 15223798 10.1167/iovs.03-1413

[32] Lee J, Cho H, Kwon I. Phase separation of low-complexity domains in cellular function and disease. Exp Mol Med. 2022;54(9):1412–22. 36175485 10.1038/s12276-022-00857-2 PMC9534829

[33] Zhou X, Sumrow L, Tashiro K, et al. Mutations linked to neurological disease enhance self-association of low-complexity protein sequences. Science. 2022;377(6601):eabn5582. 35771920 10.1126/science.abn5582 PMC9610444

[34] Polgár L. The catalytic triad of serine peptidases. Cell Mol Life Sci. 2005;62(19–20):2161–72. 10.1007/s00018-005-5160-x 16003488 10.1007/s00018-005-5160-x PMC11139141

[35] Gutteridge A, Thornton JM. Understanding nature’s catalytic toolkit. Trends Biochem Sci. 2005;30(11):622–9. 10.1016/j.tibs.2005.09.006 16214343 10.1016/j.tibs.2005.09.006

[36] Garcin C, Straube A. Microtubules in cell migration. Essays Biochem. 2019;63(5):509–20. 31358621 10.1042/EBC20190016 PMC6823166

[37] Chakraborti S, Natarajan K, Curiel J, Janke C, Liu J. The emerging role of the tubulin code: from the tubulin molecule to neuronal function and disease. Cytoskeleton (Hoboken). 2016;73(10):521–50. 26934450 10.1002/cm.21290

[38] Poirier K, Saillour Y, Bahi-Buisson N, et al. Mutations in the neuronal ß-tubulin subunit TUBB3 result in malformation of cortical development and neuronal migration defects. Hum Mol Genet. 2010;19(22):4462–73. 20829227 10.1093/hmg/ddq377 PMC3298850

[39] Ti SC, Pamula MC, Howes SC, et al. Mutations in human tubulin proximal to the kinesin-binding site alter dynamic instability at microtubule plus- and minus-ends. Dev Cell. 2016;37(1):72–84. 27046833 10.1016/j.devcel.2016.03.003 PMC4832424

[40] Minoura I, Takazaki H, Ayukawa R, et al. Reversal of axonal growth defects in an extraocular fibrosis model by engineering the kinesin-microtubule interface. Nat Commun. 2016;7:10058. 26775887 10.1038/ncomms10058 PMC4735607

