# Supplementary material for: Clinical and genetic characteristics of Chinese patients with congenital fibrosis of the extraocular muscles
Source: Orphanet J Rare Dis. 2024 Aug 15;19:300. doi: 10.1186/s13023-024-03206-w (PMC11325808; doi:10.1186/s13023-024-03206-w)
Supplement: Supplementary file 2 — Supplementary Material 2 [file 13023_2024_3206_MOESM2_ESM.docx]

Supplementary Table2. Gene list of the designed panel (GenCap Capture Kit).

| *ACTA1* | *ACTB* | *ADAMTS2* | *ADGRG1* | *AFG3L2* | *ALOX12B* | *ALOXE3* | *ALPK3* |
| --- | --- | --- | --- | --- | --- | --- | --- |
| *ALX3* | *ALX4* | *ANO3* | *ARID1A* | *ASPH* | *ASXL1* | *ATAD3A* | *ATP6V0A2* |
| *ATP6V1A* | *ATR* | *ATRIP* | *ATXN3* | *BCAP31* | *BDNF* | *BMP2* | *BRAF* |
| *C12orf57* | *CA8* | *CDC42* | *CDH11* | *CENPJ* | *CEP152* | *CEP63* | *CFL2* |
| *CHMP1A* | *CHN1* | *CHRNG* | *CHST14* | *CLDN16* | *COL12A1* | *COL1A1* | *COL25A1* |
| *COL3A1* | *COL6A1* | *COL6A2* | *COL6A3* | *COMT* | *CPLX1* | *CREBBP* | *CRYAA* |
| *CTBP1* | *CTCF* | *CYP4F22* | *DCN* | *DDHD2* | *DHCR7* | *DHODH* | *DKC1* |
| *DPYD* | *DRD5* | *DSE* | *EDNRB* | *ELN* | *EMC1* | *EMD* | *EP300* |
| *EPHA2* | *ESCO2* | *EXT2* | *FANCA* | *FANCB* | *FBN1* | *FGF10* | *FGF14* |
| *FGFR2* | *FGFR3* | *FHL1* | *FLNA* | *FMR1* | *FOXG1* | *FOXL2* | *FSCN1* |
| *GATA1* | *GBA* | *GDNF* | *GJC2* | *GLI3* | *GLIS3* | *GPR143* | *GRID2* |
| *GRM1* | *GTF2E2* | *HACE1* | *HDAC8* | *HERC2* | *HHAT* | *HRAS* | *IFIH1* |
| *IGF1* | *IRX5* | *ITGA7* | *KCNA1* | *KCNH1* | *KCNJ2* | *KCNMA1* | *KCTD1* |
| *KDM5C* | *KDM6A* | *KDM6B* | *KIF11* | *KIF21A* | *KMT2D* | *KRAS* | *KRT14* |
| *KRT16* | *KRT5* | *LAMB2* | *LETM1* | *LIPN* | *LRP2* | *MAGEL2* | *MAP2K1* |
| *MAP2K2* | *MAPK1* | *MAPRE2* | *MBD5* | *MCOLN1* | *MECP2* | *MEIS2* | *MPLKIP* |
| *MTM1* | *MTRFR* | *MYH7* | *NAA10* | *NBAS* | *NCAPG2* | *NDN* | *NDUFA1* |
| *NDUFB11* | *NDUFS1* | *NECTIN1* | *NELFA* | *NF1* | *NGLY1* | *NHP2* | *NIPBL* |
| *NOG* | *NONO* | *NOP10* | *NOTCH3* | *NR2F1* | *NRAS* | *NSD2* | *NUBPL* |
| *NYX* | *OCA2* | *OSGEP* | *OVOL2* | *PABPN1* | *PACS1* | *PAH* | *PARN* |
| *PAX3* | *PHOX2A* | *PIEZO2* | *PIGQ* | *PIK3R1* | *PMM2* | *POGZ* | *POLH* |
| *POLR1C* | *POLR3A* | *PQBP1* | *PRKAR1A* | *PRSS56* | *PTCH1* | *PTCH2* | *PTEN* |
| *RERE* | *RIPK4* | *RNF7* | *RPL10* | *RS1* | *RTEL1* | *SALL4* | *SELENON* |
| *SEMA3E* | *SETD5* | *SKI* | *SLC19A3* | *SLC1A2* | *SLC24A5* | *SLC25A46* | *SLC26A1* |
| *SLC2A10* | *SLC39A13* | *SMAD3* | *SMO* | *SNRPN* | *SON* | *SOX5* | *SPECC1L* |
| *SPG7* | *SPR* | *SPTBN2* | *SYNE2* | *TAF1* | *TBL1XR1* | *TBX1* | *TBX15* |
| *TCOF1* | *TERC* | *TFAP2A* | *TGFB3* | *TGFBR1* | *TGFBR2* | *TGM1* | *TH* |
| *THOC6* | *TNNT1* | *TP63* | *TPM2* | *TPM3* | *TTN* | *TUBB3* | *TWIST1* |
| *TYMP* | *TYRP1* | *UBE3A* | *UBE3B* | *UROS* | *USP9X* | *VLDLR* | *WAC* |
| *ZEB2* | *ZIC2* | *ZSWIM6* |  |  |  |  |  |

Supplementary Table 3 PCR primers used for Sanger sequencing to validate Next Generation Sequencing results

| **Gene** | **Primer name** | **Primer sequence** |
| --- | --- | --- |
| *KIF21A* | Exon20F1  Exon20R1 | GAGAATGAGTCAACCTTTGCTG  CGGCCAAGAAGATTACATCAG |
|  | Exon20F2  Exon20R2 | CCATTTGGAAGAAACCTTCTGA  TGCACTGCCAAATAATGAGC |
|  | Exon29F  Exon29R | TTCACCTCCTTCTTCCCCAC  CAAGAGTTCACACGTCAGGC |
| *TUBB3* | Exon4F1  Exon4R1 | ATGTCCTCCACCTTCATCGG  TCGTACATCTCGCCCTCTTC |
|  | Exon4F2  Exon4R2 | ACCATGAGCGGAGTCACC  CATCGAACATCTGCTGGGTG |
|  | Exon4F3  Exon4R3 | CTTCTTCATGCCCGGCTTC  CTGTTCTTGCTCTGGATGGC |
